# Uniaxial orientation of β-chitin nanofibres used as an organic framework in the scales of a hot vent snail

**DOI:** 10.1098/rsif.2022.0120

**Published:** 2022-06-01

**Authors:** Noriyuki Isobe, Chong Chen, Kazuho Daicho, Tsuguyuki Saito, Dass Bissessur, Ken Takai, Satoshi Okada

**Affiliations:** ^1^ Biogeochemistry Research Center, Research Institute for Marine Resources Utilization (MRU), Japan Agency for Marine-Earth Science and Technology (JAMSTEC), 2-15 Natsushima-cho, Yokosuka, Kanagawa 237-0061, Japan; ^2^ Institute for Extra-cutting-edge Science and Technology Avant-garde Research (X-STAR), Japan Agency for Marine-Earth Science and Technology (JAMSTEC), 2-15 Natsushima-cho, Yokosuka, Kanagawa 237-0061, Japan; ^3^ Department of Biomaterial Sciences, Graduate School of Agricultural and Life Sciences, The University of Tokyo, 1-1-1 Yayoi, Bunkyo-ku, Tokyo 113-8657, Japan; ^4^ Department for Continental Shelf, Maritime Zones Administration and Exploration, Prime Minister's Office, 2nd Floor, Belmont House, 12 Intendance Street, Port Louis 11328, Mauritius

**Keywords:** chitin, uniaxial orientation, scaly-foot snail

## Abstract

Organisms use various forms and orientations of chitin nanofibres to make structures with a wide range of functions, from insect wings to mussel shells. Lophotrochozoan animals such as snails and annelid worms possess an ancient ‘biomineralization toolkit’, enabling them to flexibly and rapidly evolve unique hard parts. The scaly-foot snail is a gastropod endemic to deep-sea hydrothermal vents, unique in producing dermal sclerites used as sites of sulfur detoxification. Once considered to be strictly proteinaceous, recent data pointed to the presence of chitin in these sclerites, but direct evidence is still lacking. Here, we show that β-chitin fibres (approx. 5% of native weight) are indeed the building framework, through a combination of solid-state nuclear magnetic resonance spectroscopy, wide-angle X-ray diffraction, and electron microscopy. The fibres are uniaxially oriented, likely forming a structural basis for column-like channels into which the scaly-foot snail is known to actively secrete sulfur waste—expanding the known function of chitinous hard parts in animals. Our results add to the existing evidence that animals are capable of modifying and co-opting chitin synthesis pathways flexibly and rapidly, in order to serve novel functions during their evolution.

## 1. Introduction

Chitin is a biopolymer produced by a wide range of organisms and in a great variety of forms, such as mesh sheets and rods made of elementary crystalline nanofibres [1,2]. Natural chitin nanofibres are arranged in a variety of orientations: uniaxial orientation (e.g. squid pen and crab tendon), biaxial orientation (e.g. siboglinid tubeworms), twisted plywood stack (e.g. lobster carapace), and isotropic orientation (e.g. peritrophic membranes in arthropods, annelids and tunicates, and mussel shells) [3–7]. These chitinous architectures facilitate the efficient growth of inorganic domains [8] and serve as a backbone for the secretion of inorganic (calcium carbonate or apatite) and organic (protein) compounds [9–12].

Inhabiting ‘extreme environments' such as deep-sea hot vents requires unique adaptations by the organisms, including the evolution of hard parts that may have interesting properties. Lophotrochozoan animals such as molluscs (such as snails, clams, and squids) and annelids (such as polychaete worms and earthworms) possess an ancient ‘biomineralization toolkit’ that can be traced to late Ediacaran or early Cambrian [13]. Previous studies indicate that by dynamically and flexibly modifying the expression patterns of genes contained in this toolkit, these animals are capable of rapidly evolving unique chitinous hard structures during their evolution, in relatively short periods of time [14–16]. In many cases, these hard parts are chitinous and animals also modify and co-opt chitin synthesis pathways from existing hard parts to produce the new morphological features—often with very different appearances and novel functions [16]. Unravelling the chemical and structural properties of chitinous scaffolds in such hard parts is expected to provide new insights to their uses [17–19].

One significant example is the dermal sclerites in the scaly-foot snail (*Chrysomallon squamiferum* [20]) found in a number of deep-sea hot vents in the Indian Ocean, the only gastropod mollusc possessing true dermal scales. These imbricating scales were first thought to have a defensive purpose [21,22], but recently their true function was revealed to be a detoxification site used by the animal to actively secrete sulfur waste [23], most likely from their sulfur-oxidizing endosymbiont [24]. The scales are secreted from the base (where they are attached to the foot), and growth is accretionary in the longitudinal direction, similar to human fingernails. The sulfur is secreted in column-like channels within the scales, and in localities where the vent fluid is iron-rich, the sulfur columns react with abiotically diffusing iron ions to form iron sulfide nanoparticles [23], including pyrite and greigite [25]. Those from the iron-rich Kairei vent field, for example, become metallic black in appearance from the crystallized iron sulfide particles, whereas those from the iron-poor Solitaire vent field lack iron sulfide minerals and are whitish in colour [25].

Originally, the scales were considered to be proteinaceous [21], but a recent genomic study has revealed high expression of genes related to chitin secretion-like chitin synthase and chitin-binding peritrophin-A in the scale-secreting epithelial cells [14], suggesting that it is in fact a protein–chitin composite similar to other biological structures such as molluscan shells and arthropod exoskeleton [26]. Gene expression analyses of the scale-secreting tissue revealed that the animal has likely co-opted chitin synthesis pathways from shell secretion in order to serve as a framework for the scales, although the expression patterns of the ‘biomineralization toolkit’ genes were completely different [14]. Together, these findings indicate the sclerite of the scaly-foot snail is likely actually composed of an unusual chitinous framework, but its chitinous nature has not been confirmed with direct evidence. Chitinous hard parts are typically tools used for predation, defence, locomotion, or structural support; using such hard parts as a sulfur detoxification site is unknown among animals. Here, based on spectroscopic and microscopic observations of the scales, we examined the presence of chitin fibres and their orientation in this unique biological structure, in order to substantiate its position as a chitinous novelty that expands the known functions of chitinous hard parts in animals.

## 2. Methods

### 2.1. Materials

**Figure 1.**
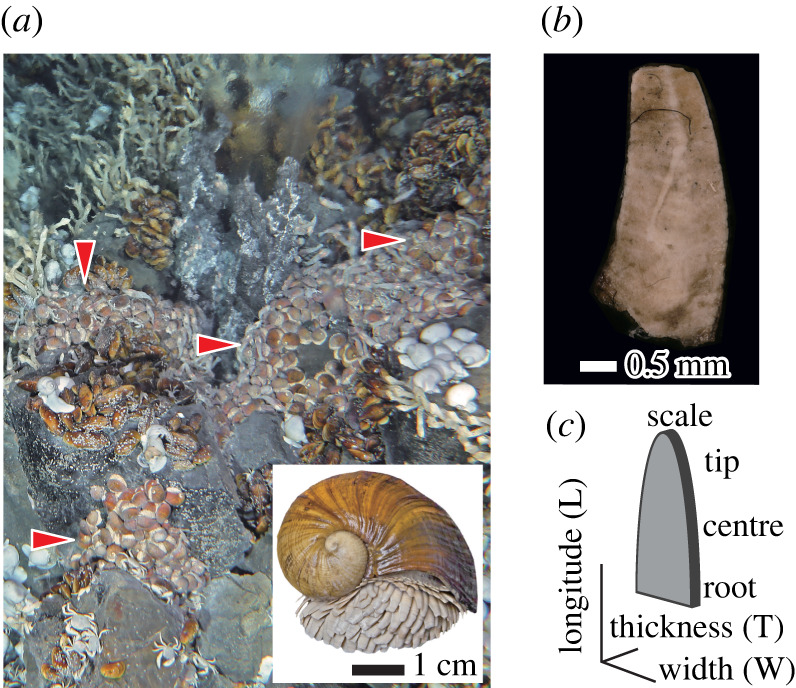
Scaly-foot snail in Solitaire hydrothermal vent field and its scales. (*a*) Aggregations of the scaly-foot snail (red arrows) *in situ* in Solitaire field. An adult individual of the scaly-foot snail is shown in the inset. (*b*) Scale in the native state. (*c*) Schematic representation of scale.

Scaly-foot snails with whitish sclerites in native, unaltered condition were collected from the iron-poor Solitaire vent field (19°33.41′ S, 65°50.89′ E, 2606 m depth, February 2013; figure 1*a*) using a suction sampler mounted on the deep submergence vehicle (DSV) *Shinkai 6500* during the R/V *Yokosuka* cruise YK13-02. The snails (inset in figure 1*a*) were immediately frozen in a –80°C freezer upon recovery on-board the research vessel, until the scales were removed in the laboratory. The average water temperature inside the scaly-foot snail colony was 27 ± 6°C [27].

All reagents used were purchased from Nacalai tesque Inc., Fuji Film Wako Pure Chemical Co., Kanto Chemical Co. Inc., or Tokyo Kasei Industry Co. Ltd, and used as received. For water source during the experiments, we used deionized water from a Merck Milli-Q Integral 5.

### 2.2. Deproteination of scaly-foot snail's scales

Three frozen scaly-foot snails were defrosted at room temperature and the whitish scales were removed using tweezers (figure 1*b*,*c*). The scales were immersed in 70% ethanol and sonicated for 3 min thrice to remove surface adsorbents including bacteria, washed with ethanol and oven-dried at 60°C overnight, resulting in brown-coloured dried scales. These dried scales still contained some invaginating foot tissue, and therefore the region near the root was trimmed off by a scalpel.

Proteins were removed from the pre-cleaned scales as reported [28]. In a glass beaker, 524.8 mg of dried scales were immersed in 7% HCl aq (20 ml) overnight at room temperature. The colourless solution was removed and washed with deionized water, and 20 ml of 1 M NaOH aq was added to it. The solution became yellow after 4 h of immersion, after which the solution was replaced with fresh NaOH aq. The replacement was performed thrice, once every 12 h, resulting in transparent scales in the end. The scales were then immersed in water for 3 h, washed again with clean water, dehydrated in a graded ethanol series (50–100%), substituted with *t*-butanol, and freeze-dried to obtain deproteinated scales (30.3 mg), which were whitish to brownish in colour. The removal of protein from white-coloured scales of the scaly-foot snail in an alkaline solution yielded transparent gels, which were sufficiently stiff for handling with tweezers, and further drying yielded dried solid scales white to brown in coloration. The scales retained their original shape but the width and height shrunk to 72 ± 6% and 65 ± 18% (average ± s.d., *N* = 6), respectively, and the total weight of the scales decreased to 5.8%. Optical micrographs were obtained using a Keyence VHX-5000 equipped with a VH-ZST zoom lens at each step of the chemical treatment. As a reference, a squid pen (the gladius) of a Japanese flying squid (*Todarodes pacificus*) was deproteinated in the same manner.

### 2.3. Scanning electron microscopic observation

Scanning electron microscopy (SEM) imaging with energy-dispersive X-ray spectroscopy (EDS) analyses were performed on a Helios G4 UX (Thermo Fisher Scientific) equipped with an Octane Elite Super EDS detector (AMETEK) in Japan Agency for Marine-Earth Science and Technology (JAMSTEC). The SEM–EDS was operated at a landing voltage of 1 kV for SEM imaging and 20 kV for elemental analyses without any conductive coating. A dried, deproteinated scale was mounted on an aluminium pin stub using a piece of double-sided carbon tape (Okenshoji Inc.). The mounted scale was fractured using tweezers to expose its cross-section.

### 2.4. Spectroscopic mapping

Microscopic transmission Fourier-transform infrared (FT-IR) spectroscopy mapping images were collected on a JASCO FT/IR-6200 type-A spectrometer equipped with an IRT-7000 IR microscope. The deproteinated and dried scales were placed on a fluorite plate and spectra were collected in a spectral range from 1000 to 4000 cm^–1^ with a spatial pixel resolution of 50 × 50 µm. Spectra were recorded at a spectral resolution of 4 cm^–1^ with 128 times of integration.

### 2.5. Wide-angle X-ray diffraction and small-angle X-ray scattering

Wide-angle X-ray diffraction (WAXD) experiment was performed using a NANO VIEWER (Rigaku Japan) at 40 kV and 40 mA or a Nanopix (Rigaku Japan) at 40 kV and 30 mA with monochromatized and collimated Cu K*α* radiation (*λ* = 1.548 Å). Small-angle X-ray scattering (SAXS) was carried out with synchrotron radiation at the BL40B2 beamline of SPring-8 (Hyogo, Japan), using a native scaly-foot snail scale mounted on a goniometer head. The X-ray (*λ* = 1.0 Å) was irradiated for 10 s. The distance between the sample and the imaging plate (3191.1 mm) was calibrated using silver behenate powders (*d* = 5.838 nm) [29]. The obtained WAXD diagram was converted to a one-dimensional azimuthal profile using Rigaku 2DP software. The degree of orientation (DO) was calculated from the azimuthal profile of the reflection at 2*θ* = 8.8° using the following equation: DO = (180° − FWHM)/(180°), where FWHM is the full width at half maximum.

### 2.6. Solid-state nuclear magnetic resonance

Native whitish scales were ground to powder in a ceramic mortar. Deproteinated scales were difficult to grind and were instead cut into small flakes by scissors and scalpels. ^13^C cross-polarization (CP) magic-angle spinning (MAS) solid-state nuclear magnetic resonance (NMR) spectra were acquired on a JEOL JNM-ECAII 500 spectrometer equipped with 3.2 mm HXMAS probe and ZrO_2_ rotor at 125.77 MHz. The 90° proton decoupler pulse width, contact time, relaxation delay and spinning frequency were 2.5 µs, 2 ms, 5 s and 15 kHz, respectively.

### 2.7. Observation of gold-stained scale

A piece of the native, whitish scale from the scaly-foot snail was cut into two pieces along with the longitudinal axis to enhance the chemical treatment of the internal surface, following the conventional procedure of periodic acid–methenamine silver (PAM) staining of polysaccharides with gold chloride in an Eppendorf tube [30]. The colour of the scales changed from cream to red after immersion in thiosemicarbazide solution. After methenamine–silver nitrate treatment, the pieces were immersed in sodium tetrachlorogold solution for 5 min followed by washing with Na_2_S_2_O_5_ solution for 5 min and three times in water. The resulting wet sample was dehydrated in a graded ethanol series and embedded in epoxy resin (TAAB) following Luft's method [31]. The resin blocks were trimmed with a razor and sliced to 100 nm and 1 µm thick sections using an ultramicrotome (Ultracut S or EM UC7, Leica) with a diamond knife (45°, Diatome), and collected on a formvar-supported Cu/Rh grid mesh.

The 1 μm thick sections were imaged in SEM–EDS using a scanning transmission electron microscopy (STEM) detector, and the 100 nm thick sections were imaged using a transmission electron microscope (TEM). In the STEM imaging, the grid was fixed with an aluminium jig, resulting in strong background signals of aluminium. TEM imaging was performed on Tecnai G2 20 (Thermo Fisher Scientific) operating at 200 kV and equipped with a bottom-mounted 2 k × 2 k Eagle charge-coupled device (CCD) camera (Thermo Fisher Scientific). Image montage was manually prepared using Affinity Photo 1.9.1 and sizes were measured and analysed using ImageJ 2.1.0/1.53c [32].

## 3. Results

### 3.1. Identification of chitin in the native scaly-foot snail scale

**Figure 2.**
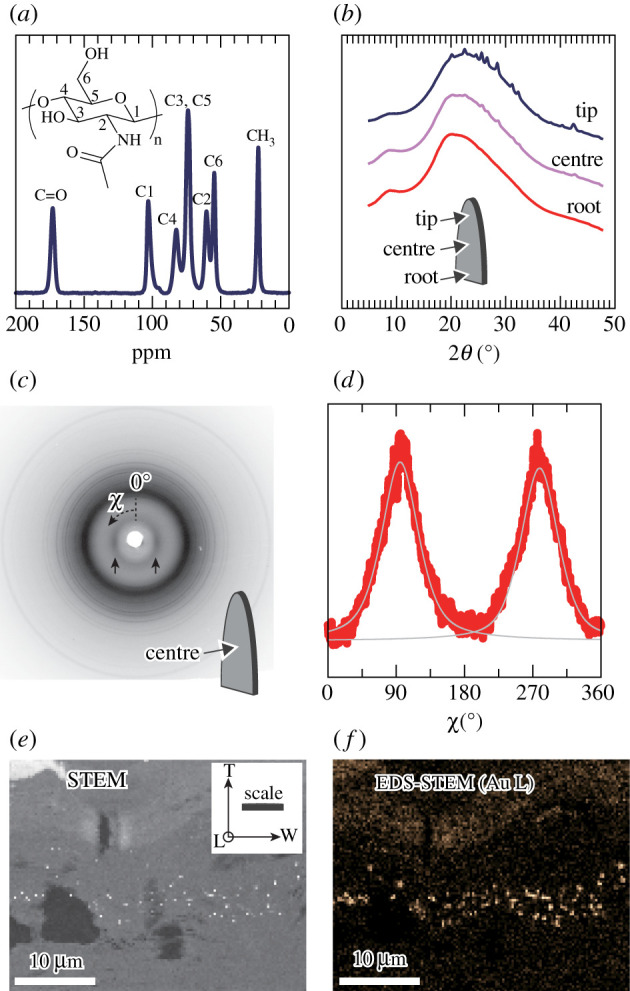
Characterization of the crystalline component of native scaly-foot snail's scale. (*a*) ^13^C CP/MAS NMR spectrum of the native scale. (*b*) WAXD patterns of native scaly-foot snail scales at different positions. (*c*) Diffraction pattern of a native scale taken at the centre in the through-view direction as depicted in schematic image. Reflections at 2*θ* = 8.8° of chitin are indicated with arrows, and azimuthal angle *χ* is indicated. (*d*) Azimuthal plot of reflections at 2*θ* = 8.8° of chitin. The line shape was fitted by a Gaussian function represented by solid lines in grey, and the full width at half maximum was calculated. (*e*) STEM image of thin section of PAM-stained scale sectioned perpendicular to the longitudinal direction. The orientation of the scale is noted in the inset by letters and arrows: L, longitudinal; W, width; H, height. (*f*) STEM–EDS (Au L) mapping of a semi-thin section using another thin section of (*e*).

First, the presence of crystalline chitin in the native scale of scaly-foot snail was detected via solid-state ^13^C CP/MAS NMR spectroscopy (figure 2*a*), the pulse sequence of which was optimized for the detection of crystalline polysaccharides such as cellulose [33]. The NMR spectrum was in good agreement with that of chitin: chemical shifts at 22.4–23.5 (acetyl CH_3_), 54.5–55.7 (C2), 60.2–61.4 (C6), 73.9–75.0 (C3 and C5), 102.8–104.0 (C1) and 173.0–174.0 (C=O) ppm [34–37]. The signals originating from proteins were not noticeable in the spectrum of the native scale, despite the high protein content of about 95% (see Methods section). Such absence of signals was also observed in the ^13^C CP/MAS spectrum of the chitinous tube of a deep-sea siboglinid tubeworm. It is possible that efficient magnetization transfer to carbon atoms in the proteins in the scale may not have taken place with the pulse sequence used in this study. However, the reason behind their absence remains unclear [34].

To further understand the crystalline structure and the alignment of chitin fibres in the scales, WAXD (figure 2*b–d*) was performed. Diffraction profiles of the native scale at different positions (figure 2*b*) showed broad peaks centred around 2*θ* = 8.8° and 20–25° originating from chitin, accompanied by several sharp peaks positioned between 2*θ* = 20° and 45°, all indexed with barite crystal (BaSO_4_), one of the possible minerals naturally precipitating from the Solitaire vent fluid [38]. As the scales grow from the root, the distal parts near the tip are older and have been exposed longer to vent fluids in the environment [27]; this enrichment pattern is therefore in agreement with the secretion mechanism of the scale [39]. Due to the broad nature of peaks from chitin in the native-state scales, it is difficult to distinguish the chitin crystal allomorph in the native scale, namely *α* or β [40,41], from these data alone.

The two-dimensional X-ray diffraction diagram (figure 2*c*) displays the alignment of chitin. The reflections from chitin crystal were observed as equatorial reflection spots in the native scale (arrows in figure 2*c*), whereas all the barite-originated reflections were present as ring-shaped powder diffraction pattern. The azimuthal profile of the reflections centred at 2*θ* = 8.8° (figure 2*d*) clearly shows the unidirectional alignment of chitin nanofibres along the secretion direction with a DO of approximately 70%, which was calculated from full width at half maximum of the azimuthal profile.

The spatial distribution of the chitin fibres within the native scale was further imaged in STEM using the PAM staining method, in which chitin fibres were stained with *in situ* generated gold nanoparticles (figure 2*e*,*f*). The imaging was performed on thin sections sliced perpendicular to the longitudinal axis of the scale. The white spots in STEM image and orange spots in EDS mapping image represent the gold particles bound to chitin. The gold particles were located in close proximity to each other, implying an aggregated layer structure of chitin with a thickness of several tens of micrometres.

Additionally, SAXS measurement of the native scale revealed uniaxial alignment on a larger scale (up to 200 nm; electronic supplementary material, figure S1). The two-dimensional X-ray scattering diagram showed uniaxial orientation along the secretion direction, evidenced by the ellipsoidal shape scattering pattern (electronic supplementary material, figure S1a). The diffraction profile obtained from the equatorial direction (*I*(*q*) versus *q*; electronic supplementary material, figure S1b) and Kratky plot thereof (*q*^2^ · *I*(*q*) versus *q*; electronic supplementary material, figure S1c) exhibited two peaks at 0.0067 and 0.0115 Å^−1^, indicating the periodicity of 90 and 55 nm, respectively. These do not arise from the chitin nanofibres themselves but from the previously reported uniaxially aligned sulfur-rich columns in the scale, mostly 100–150 nm in diameter with some at 50 nm or less [17].

### 3.2. Characterization of chitin in the deproteinated scaly-foot snail scale

**Figure 3.**
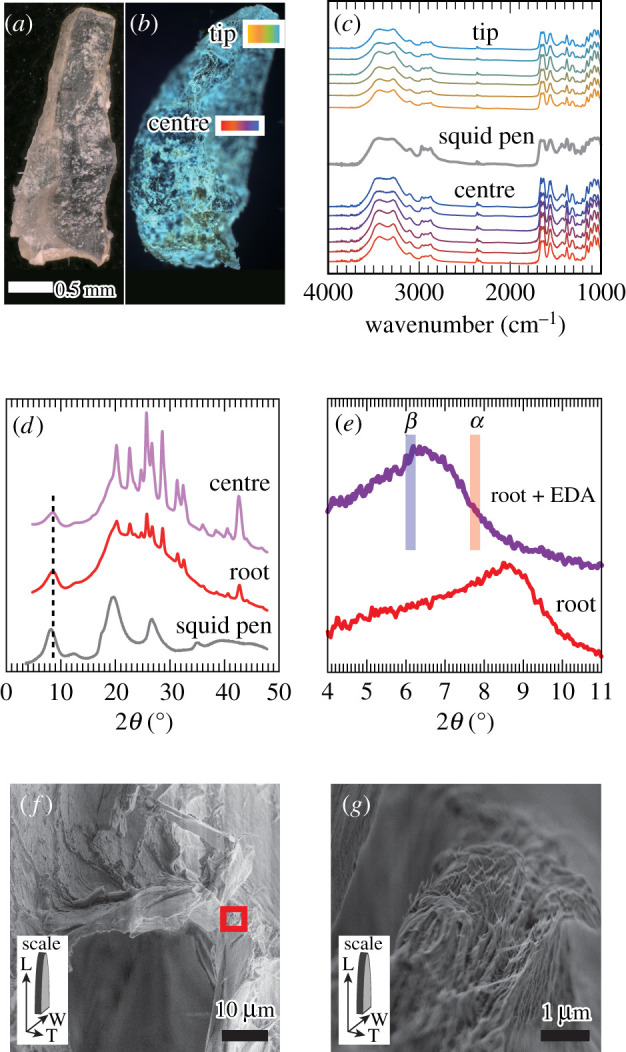
Characterization of the chitin in the deproteinated scale of scaly-foot snail. (*a*) Photograph of a deproteinated scale. (*b*) Optical micrograph of the deproteinated scale showing the analysed regions (rectangles framed in white). (*c*) IR spectra at the tip and centre of the scale, as well as the pen of the squid *Todarodes pacificus* (grey) shown as a reference. The colours indicate the position within the white rectangles in (*b*). (*d*) WAXD profiles of deproteinated scales at the root (red) and centre (purple) of the scale. A profile of a purified squid pen from *T. pacificus* (grey) is shown as a reference. Reflection from (010) plane of β-chitin monohydrate is indicated with dotted line. (*e*) WAXD profile of deproteinated scale soaked in EDA. A profile of deproteinated scales at the root is shown as a reference. Reflections from (010) plane of type II of β-chitin EDA complex and (020) plane of α-chitin EDA complex are indicated with lines in blue and red, respectively. (*f*) SEM image of the deproteinated scale. Red rectangle is the observed region shown in (*g*). (*g*) High-magnification SEM image of the deproteinated scale. The orientation of the scale is noted in inset letters and arrows: L, longitudinal; W, width; H, height.

To further analyse chitin in the scale, inorganic and abundant protein components were removed through a mild acid–base treatment (figure 3*a*). Transmission FT-IR mapping of the deproteinated scale was performed to investigate the spatial homogeneity of chitin fibres along the width direction (figure 3*b*,*c*). Characteristic bands at approximately 3450 (OH stretch), approximately 3280 (N–H stretch), 1620–1670 (amide-I) and 1555 cm^–1^ (amide-II) were found, which matched well with the spectra of chitin from the squid pen [42,43]. The IR spectral patterns at different positions were similar with regards to their line shape, relative intensity among peaks, and peak position, indicating the deproteinated scale principally consists of chitin.

The solid-state NMR spectrum of the deproteinated scale was almost identical to that of the native-state scale (electronic supplementary material, figure S2), indicating that after the mild acid–base treatment the chitin structure was maintained. The degree of acetylation (DA) was calculated from the spectrum of the native scale to be 83% by DA (%) = (*I*_acetyl CH3_/*I*_C1_) × 100, where *I*_acetyl CH3_ and *I*_C1_ denote relative integrals of corresponding carbon signals [44], which is in accordance with that of chitin in insects and molluscs [45,46].

In the WAXD profiles (figure 3*d*), a characteristic peak stood out at 2*θ* = 8.5° upon the removal of proteins. This reflection corresponds to a *d*-spacing of 10.4 Å, which is indexed as (010) plane of the monohydrate of β-chitin [36,47]. The formation of hydrates is one of the unique characteristics in β-chitin, with which one can distinguish the chitin allomorphs [48]. Owing to the absence of hydrogen bonds between hydrophobically stacked molecular sheets in the β-chitin crystal, β-chitin can incorporate water molecules between molecular sheets upon hydration, leading to the expansion of *d*-spacing along [010] direction from 9.2 Å (2*θ* = 9.6°, anhydrous [41,47,49]) to 10.4 Å (2*θ* = 8.5°, monohydrate [36,47]) or 11.1 Å (2*θ* = 8.0°, dihydrate [36,47,50]), while no such peak shift is observed when α-chitin is hydrated. However, this method is not effective for poorly crystalline samples (small crystal size or low crystallinity), as the width of the peak increases upon hydration and makes it difficult to detect the hydration-induced peak shift. Recently, we developed a novel method [51] based on the incorporation of ethylenediamine (EDA) into chitin crystals [52–54]: both *α*- and β-chitin show a clear peak shift originating from the incorporation of EDA molecules between hydrophobically stacked molecular sheets, but the *d*-spacing is wider in the case of β-chitin due to the absence of hydrogen bonds between molecular sheets, exhibiting *d*-spacing of 14.4 Å (2*θ* = 6.1°, (010) plane of type II β-chitin EDA complex [53]) and 11.4 Å (2*θ* = 7.8°, (020) plane of α-chitin EDA complex [52]). With this EDA complexation method, the lowest peak position of the deproteinated scale shifted to 2*θ* = 6.2° (figure 3*e*), confirming that chitin in the scaly-foot snail's scales is indeed the β form. This β-chitin exists in the form of nanofibres with a diameter of 7–50 nm observed in the high-resolution SEM images of the mechanically fractured cross-section (figure 3*f*,*g*) showing the bundled nanofibrous structure, mostly oriented along the longitudinal axis. Note that the drying process inevitably caused aggregation of adjacent fibres, and the fibre diameter does not necessarily reflect the true diameter of chitin fibres within native-state scales.

It should be noted that the peaks from barite crystals stood out more strikingly in WAXD profiles after the removal of proteins, due to the insoluble nature of barite crystals. SEM–EDS analyses of the deproteinated scale (electronic supplementary material, figure S3) revealed that they were carbonaceous, with white powders of barite containing other alkaline earth metals attached, difficult to remove through chemical treatment without damaging the organic material. The presence of barite particles implies that our measured weight of the deproteinated scale is a slight overestimation and the weight ratio of the organic fibres may be less than 5% of the native scale.

## 4. Discussion

Our results show that chitin is the central scaffolding material used in the scales of scaly-foot snail, unambiguously confirming that the scale is a protein–chitin composite and not purely proteinaceous as previously thought. The whole-genome assembly of the snail [14] revealed an expansion of gene families related to chitin-related metabolic processes and chitin binding, in line with our results. The snail possesses a diverse range of chitin synthases ranging from type I to four groups (A–D) of type II, with a paralogue of the type-IIC chitin synthase being especially dominant in the scale-secreting epithelium [14]. Our results confirm this chitin synthase does indeed produce chitin to build the organic framework. Different types of chitin synthase were highly expressed in the scale-secreting epithelial cells compared to the shell-secreting mantle cells, suggesting the scales may produce chitin in a different manner from the shell.

Linear polysaccharides such as cellulose and chitin have a molecular direction: one end is the reducing end and the other is the non-reducing end. Chitin crystals are categorized into α or β according to this molecular directionality. While the molecular direction of adjacent chitin chains is opposite in α-chitin (anti-parallel packing), all chitin molecules align in the same direction in β-chitin (parallel packing). It is known that the extension of β-chitin always occurs at the non-reducing end [55,56]. The production of β-chitin in scaly-foot snail scales is in accordance with the accretionary secretion mechanism of the scale from the root: chitin chains are secreted from one end (the scale-secreting epithelium at the root), resulting in the same molecular direction for all fibres and generating uniaxially oriented β-chitin. In the case of the scaly-foot snail scales, the extension must be occurring at the root, where the scale-secreting epithelium is located [23]. The scales exhibit uninterrupted sulfur-enriched columns proposed to be a detoxification site [23], and the uniaxially oriented β-chitin framework likely helps with the formation of these column-like structures.

Our results also show that about 95% of the scale is not comprised of chitin. From gene expression analyses of the scaly-foot snail [17], we know that the paralogues of the chitin-binding peritrophin-A were especially highly expressed in the scale-secreting epithelium, indicating the protein product(s) of this gene is likely a major component of the other 95%. How exactly the gene product(s) interact with chitin to form the final sclerite structure, however, warrants future studies.

## 5. Conclusion

We revealed that β-chitin is a key structural component of scaly-foot snail scales, through a combination of solid-state NMR, WAXD, and electron microscopy. The weight ratio of chitin comprised approximately 5% of the scale's total dry weight, while the other 95% is likely proteinaceous. β-Chitin is present in the form of uniaxially oriented fibre bundles elongated from the root outwards in the longitudinal axis, likely acting as a structural basis for forming sulfur-enriched columns along the same axis into which the snail is known to actively secrete sulfur waste. Chitin-based hard parts serve a wide range of functions in different organisms, and here they likely assist in detoxification of sulfur waste from endosymbiosis. By confirming that the scaly-foot snail's unique sclerites are chitinous in nature, our results expand the function of chitinous hard parts and add to the evidence that animals are capable of modifying and co-opting chitin synthesis pathways flexibly and rapidly in order to serve novel functions during evolution.

## Data Availability

The datasets generated and/or analysed during the current study are available from the corresponding author on reasonable request. The data are provided in the electronic supplementary material [57].
